# An automated hip fracture detection, classification system on pelvic radiographs and comparison with 35 clinicians

**DOI:** 10.1038/s41598-025-98852-w

**Published:** 2025-05-08

**Authors:** Abdurrahim Yilmaz, Kadir Gem, Mucahit Kalebasi, Rahmetullah Varol, Zuhtu Oner Gencoglan, Yegor Samoylenko, Hakan Koray Tosyali, Guvenir Okcu, Huseyin Uvet

**Affiliations:** 1 Division of Systems Medicine, Department of Metabolism, Digestion, and Reproduction, Imperial College London, London, SW7 2AZ UK; 2 Department of Orthopedics and Traumatology, Manisa Alasehir State Hospital, 45600 Manisa, Turkey; 3 Mechatronics Engineering Department, Yildiz Technical University, 34349 Istanbul, Turkey; 4 Department of Orthopedics and Traumatology, Manisa City Hospital, 45040 Manisa, Turkey; 5 Department of Orthopedics and Traumatology, Manisa Celal Bayar University Hafsa Sultan Hospital, 45030 Manisa, Turkey

**Keywords:** Pelvis X-ray, Hip fractures, Automated fracture detection, Information technology, Biomedical engineering

## Abstract

Accurate diagnosis of orthopedic injuries, especially pelvic and hip fractures, is vital in trauma management. While pelvic radiographs (PXRs) are widely used, misdiagnosis is common. This study proposes an automated system that uses convolutional neural networks (CNNs) to detect potential fracture areas and predict fracture conditions, aiming to outperform traditional object detection-based systems. We developed two deep learning models for hip fracture detection and prediction, trained on PXRs from three hospitals. The first model utilized automated hip area detection, cropping, and classification of the resulting patches. The images were preprocessed using the Contrast Limited Adaptive Histogram Equalization (CLAHE) algorithm. The YOLOv5 architecture was employed for the object detection model, while three different pre-trained deep neural network (DNN) architectures were used for classification, applying transfer learning. Their performance was evaluated on a test dataset, and compared with 35 clinicians. YOLOv5 achieved a 92.66% accuracy on regular images and 88.89% on CLAHE-enhanced images. The classifier models, MobileNetV2, Xception, and InceptionResNetV2, achieved accuracies between 94.66% and 97.67%. In contrast, the clinicians demonstrated a mean accuracy of 84.53% and longer prediction durations. The DNN models showed significantly better accuracy and speed compared to human evaluators (*p* < 0.0005, *p* < 0.01). These DNN models highlight promising utility in trauma diagnosis due to their high accuracy and speed. Integrating such systems into clinical practices may enhance the diagnostic efficiency of PXRs.

**Supplementary Information:**

The online version contains supplementary material available at 10.1038/s41598-025-98852-w.

## Introduction

Trauma management is a race against time. Accordingly, correct management and accurate diagnosis are the most important factors for saving lives.^1^ High-quality trauma care and treatment depend not only on physicians’ experience but also on the correct use of various imaging modalities and innovative technologies such as X-ray imaging. X-ray imaging is one of the most widely used diagnostic imaging modalities worldwide for evaluating blunt trauma patients. Pelvic radiographs (PXRs) cover the pelvis and upper femur region, providing diagnostic value for orthopedic injuries in these regions, including hip fracture, pelvic fracture, hip dislocation, and other associated injuries.^2^ Hip fractures, which are the most common type of fracture for old people, are diagnosed using PXRs.^3^ However, misdiagnosis rates range from 4% and 9%, and delayed diagnosis can result in serious complications.^4^ Pelvic fractures are among the most life-threatening, with mortality rates exceeding 30% in unstable patients.^5,6^ Early diagnosis and treatment in the emergency department can prevent complications for injured patients. However, image-based diagnosis often relies on emergency physicians who first meet the patient in a chaotic and stressful emergency room. Moreover, radiologists are not always available 24/7, especially in local hospitals or rural areas. An effective computer-aided diagnostic algorithm can help to prevent misdiagnoses and provide early warnings for life-threatening complications.^7^ In recent years, deep learning methods have shown remarkable success in medical imaging applications, ranging from skin lesion classification to brain tumor segmentation.^8^

Several studies have investigated the use of deep learning algorithms, such as convolutional neural networks (CNNs), for the detection of pelvic and hip fractures from radiographic images. These studies have shown that deep learning algorithms can achieve high accuracy in identifying fractures and can provide more detailed insights into the location and severity of fractures compared to traditional methods.^9–12^ Additionally, object detection methods were applied to pelvis region analysis and fracture detection tasks. Furthermore, segmentation techniques, such as multi-scale feature fusion networks, two-step pipelines that combine segmentation and recognition, have also demonstrated high accuracy and refined localization in medical image analysis using different modalities, for orthopedic fracture detection.^13–17^ Compared to traditional image classification methods, two-step approaches were developed for different cases such as segmentation with the support of detection,^18^ detection with the support of segmentation,^19^ and object detection methods that have the advantage of providing precise localization information for hip fractures.^20–22^ We implemented a classification approach alongside detection to identify fracture regions in PXR images.

In this study, we present an automated system to localize potential fracture areas in the left and right regions and to predict fracture presence using CNN-based deep learning approaches instead of direct object detection-based systems. We combined two deep learning models based on the You Only Look Once v5 (YOLOv5) model for object detection to locate the pelvis region and pre-trained CNN models as a classifier to predict fractures. We also implemented the Contrast Limited Adaptive Histogram Equalization (CLAHE) algorithm. Finally, we measured the effectiveness of the study by comparing this deep learning system with 35 clinicians. To summarize our main contributions: (1) we developed a preprocessing pipeline for hip fracture detection and compared its performance with a simple object detection-based fracture detection system, (2) we implemented an automated cropping system for femur regions and integrated the CLAHE algorithm, and (3) we compared this system between our system and 35 clinicians of varying experience levels, including a detailed statistical analysis of prediction durations on the test dataset.

## Results

This section presents the weighted metrics obtained from the experimental evaluation of our proposed AI models. All experiments were conducted using either a holdout or 5-fold cross-validation scheme to validate the robustness of our proposed models. Accuracy, precision, F1 score, IoU, mAP and inference time values of YOLOv5 object detection model to detect fracture on non-CLAHE PXR images are 92.66%, 94.50%, 94.50%, 0.8832, 94,80%, and 4.63 s, respectively. For CLAHE applied images, accuracy, precision, F1 score, IoU, mAP and inference time values are 88.89%, 89.6%, 88.6%, 0.9033, 92.80%, and 4.43 s, respectively. For hip area detection success of YOLOv5, IoU and mAP values are 0.9255 and 96.43%, respectively. Mean accuracy, precision, F1 score, AUC score, and inference time values for MobileNetV2 on non-CLAHE images are 95.15% ± 1.10, 95.11% ± 1.13, 95.08% ± 1.12, 0.9905, and 0.81 s, respectively. MobileNetV2 performance for CLAHE images as mean average accuracy, precision, F1 score, AUC score, and inference time values are 94.66% ± 1.02, 94.86% ± 0.67, 94.69% ± 0.91, 0.9863, and 0.75 s, respectively. The mean accuracy, precision, F1 scores, AUC score, and inference time values for the Xception model on non-CLAHE images are 97.67% ± 0.53, 97.65% ± 0.55, 97.63% ± 0.55, 0.9947, and 1.06 s, respectively. The performance of Xception on CLAHE images for mean accuracy, precision, F1 score, AUC score, and inference time values are 96.01% ± 1.10, 95.98% ± 1.13, 95.94% ± 1.11, 0.9930, and 1.07 s, respectively. Mean accuracy, precision, F1 score, AUC score, and inference time values of InceptionResNetV2 for non-CLAHE images are 96.75% ± 0.42, 96.76% ± 0.41, 96.70% ± 0.43, 0.9941, and 2.47 s, respectively. InceptionResNetV2 performance for CLAHE images as mean accuracy, precision, F1 score, AUC score, and inference time values are 96.99% ± 0.23, 97.02% ± 0.25, 96.96% ± 0.25, 0.9955, and 2.33 s, respectively. All models were evaluated on the test samples using the Monte Carlo method, and they exhibited low uncertainty, as shown in Supplementary Fig. 1.

**Fig. 1:**
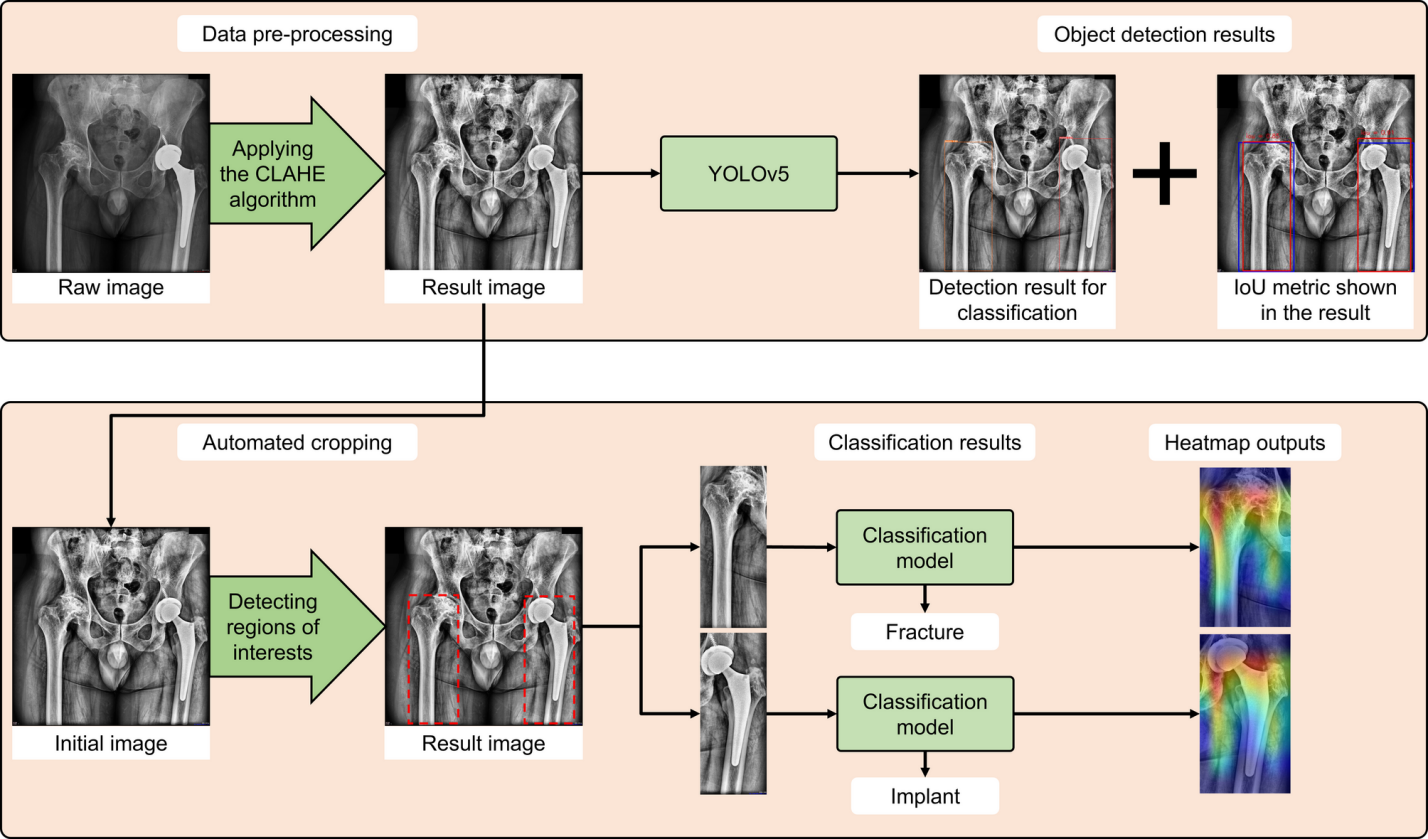
Shows an example from test dataset for object detection model and classification model with GradCAM results.

In this study, 35 clinicians were evaluated on the 326 PXR images of the test dataset. Their mean values and standard deviations of 35 clinicians for accuracy, precision, and F1 scores are 84.53% ± 15.01, 89.90% ± 9.21, and 87.13% ± 11.41, respectively. The prediction duration for 326 images of the test dataset was 16.07 ± 6.26 minues and 2.20 ± 1.58 seconds for the clinicians and AI models, respectively. The accuracy, precision, and F1 score metrics of the AI models were statistically higher than 35 clinicians (*p* < 0.0005, *p* < 0.01, and *p* < 0.0005, respectively). Figure 1 shows an example in which the model succesfully performs both straightforward object detection and hip area detection with classification model, visualized using Gradient-weighted Class Activation Mapping (GradCAM).^23^ The performance of object detection and three deep learning models and their standard deviations are presented in Table 1.

**Table 1:** Shows metrics of object detection, classification models, and 35 clinicians. Statistical significance exhibits significancy among deep learning models and 35 clinicians.

Model—data	Accuracy	Precision	${F}_{1} \text{ Score}$	AUC score	Inference time
MobileNetV2—C	94.66 ± 1.02%	94.86 ± 0.67%	94.69 ± 0.91%	0.9863	0.75 s
MobileNetV2—NC	95.15 ± 1.10%	95.11 ± 1.13%	95.08 ± 1.12%	0.9905	0.81 s
Xception—C	96.01 ± 1.10%	95.98 ± 1.13%	95.94 ± 1.11%	0.9930	1.07 s
Xception—NC	97.67 ± 0.53%	97.65 ± 0.55%	97.63 ± 0.55±	0.9947	1.06 s
InceptionResNetV2—C	96.99 ± 0.23%	97.02 ± 0.25%	96.96 ± 0.25%	0.9955	2.33 s
InceptionResNetV2—NC	96.75 ± 0.42%	96.76 ± 0.41%	96.70 ± 0.43%	0.9941	2.47 s
YOLOv5—C	88.89%	89.60%	88.60%	N/A	4.43 s
YOLOv5—NC	92.66%	94.5%	94.50%	N/A	4.63 s
35 Clinicians—NC	84.53 ± 15.01%	89.9 ± 9.20%	87.13 ± 11.41%	N/A	16.07 ± 6.26% min
Statistical significance	$p<0.0005$	$p<0.001$	$p<0.0005$	N/A	$p<0.0001$

C, CLAHE applied images used in dataset; NC,  No CLAHE applied images used in dataset.

## Discussion

In this study, we developed two models: (1) a straightforward object detection model and (2) a hip area detection model with a patch classification feature. Previous studies employing object detection approaches on X-ray images have mostly labeled the fracture or related diseases and trained deep learning models accordingly.^24^ In this study, this method was further developed, and an object detection model was trained to detect the potential fracture or implant area. The patch detected by this model on PXR images was provided to the classification model and the results were obtained. It has been shown in the literature that instead of giving the whole PXR image, giving the focused region provides higher performance. In this study, it is shown that higher performance can be obtained with patches zoomed in using automatic region detection. To further improve performance, the CLAHE algorithm, which is known to be effective in literature, was also included in the training. While the performance of the classification models increased, the performance of the object detection model decreased. Using CLAHE might unintentionally amplify noise and modify subtle features necessary for accurate detection, leading to decreased model performance. The Xception model, trained on the non-CLAHE dataset, was identified as the most effective model among those developed in this study, exhibiting best performance in detecting potential fractures or implant areas in images. The Xception model distinguishes the implant class with 100% accuracy while maintaining a low error rate in distinguishing cases with fracture class and no fracture class, emphasizing the robustness of the system and its potential for accurate clinical decision support. To increase the reliability of the study, data were collected from three different hospitals, using images obtained from different devices and locations. Both developed models were superior to 35 clinicians according to classification metrics. In addition, for a test dataset of 326 images, clinicians spent an average of 16 minutes, while the AI models can analyze hundreds of images in minutes due to their computational efficiency. To fully realize the benefits of AI in clinical practice, it is essential to address challenges such as regulatory requirements, data bias, interpretability, and clinician acceptance, ensuring safe and responsible integration of AI systems into healthcare.^25–28^

This study includes some limitations. First, the dataset was collected from different hospitals and thus from different devices, causing variations in image quality. Second, the data classified as fractures and implants in the collected images were fewer than the healthy data resulting in an imbalanced dataset. Third, analyzing a large set of images (test dataset, *N* = 326) consecutively could induce fatigue in clinicians, potentially impacting their performance. Fourth, our dataset primarily comprises older patients with hip fractures and was collected from three hospitals, which may introduce demographic biases (e.g., age, gender) that could limit the generalizability of our findings, especially for younger patients.

In conclusion, this study demonstrates the significant potential of AI-driven models in improving the accuracy and speed of diagnosing hip fractures on PXRs. Utilizing YOLOv5 for object detection and pre-trained deep neural network (DNN) architectures for classification, our models achieved accuracies between 94.66% and 97.67%, outperforming clinicians who had a mean accuracy of 84.53%. The AI models demonstrated both efficiency and greater accuracy by delivering results in a reduced amount of time. Despite a slight reduction in accuracy, the application of CLAHE preprocessing maintained robust performance. These results indicate that the integration of AI systems into medical practice can improve the accuracy and efficacy of diagnostics. Further research could explore a broader range of orthopedic injuries with subtypes of fractures and address real-world implementation challenges to fully realize the benefits of AI-assisted diagnostics in trauma care. The future research could also address mitigation of class imbalance.

## Methods

**Fig. 2:**
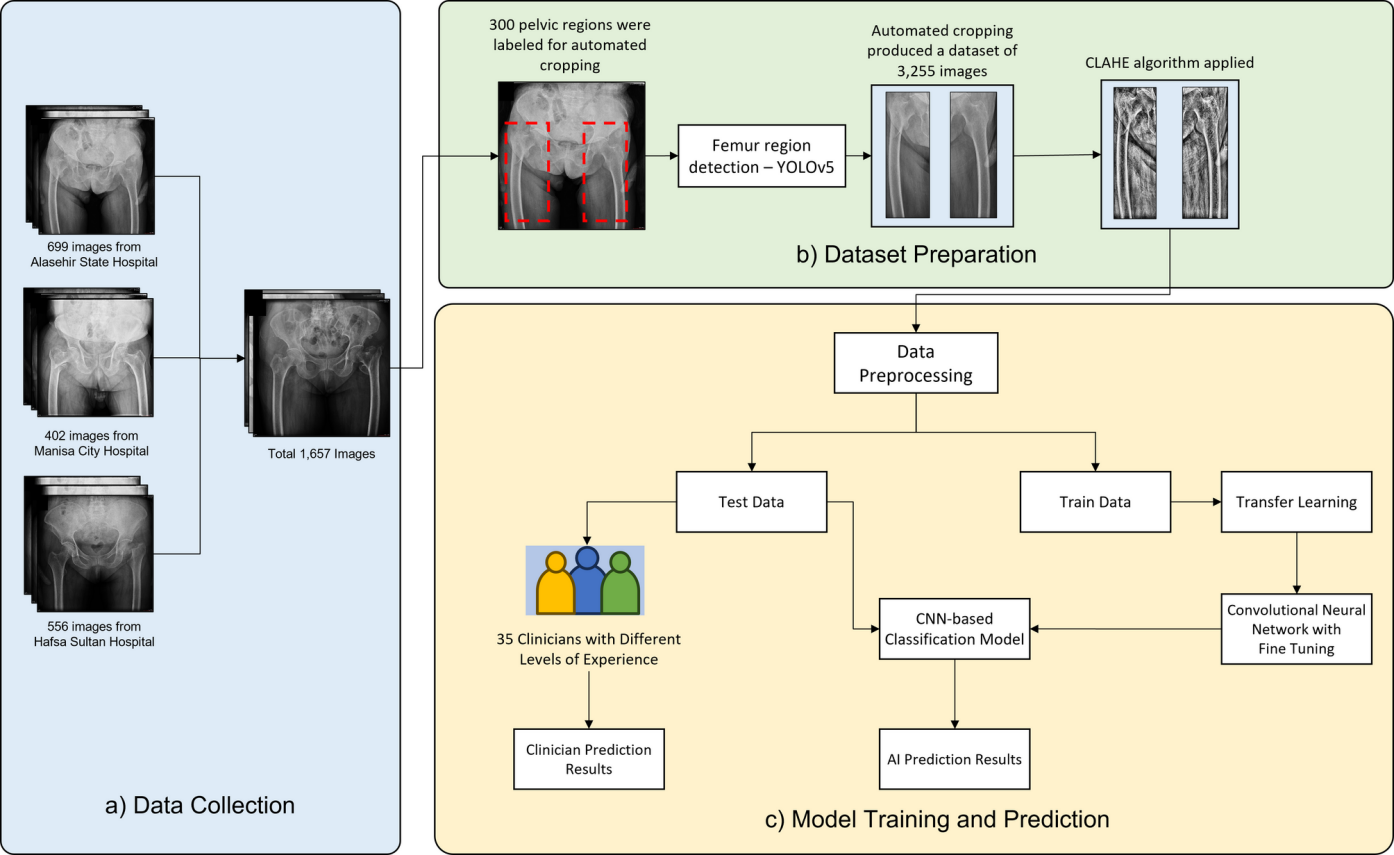
(a) We collected 1,657 images from three different hospitals. (b) We annotated the hip regions with fracture, implant, and non-fracture labels. 300 of these images were used for automated hip area detection. All of them were used to train the object detection model. The CLAHE algorithm was applied to all images, including hip patches. (c) Classification model was trained and compared with the performance of 35 clinicians.

Deep learning can autonomously extract relevant features that are difficult to identify using traditional methods, due to its neural architecture.^29^ In our study, we developed and compared two systems: the first is based on object detection for fracture identification, while the second detects the hip area, crops the relevant section, and classifies the cropped patches in an automated manner. Firstly, hip regions on PXRs were detected and annotated, and an object detection model based on YOLOv5 was trained on this dataset to detect crop regions automatically. Then, data preprocessing using the CLAHE algorithm was applied to the cropped hip images. For the classification of the patches, MobileNetV2, Xception, and InceptionResNetV2 DNN architectures were deployed using the transfer learning method. Finally, the three deep learning models were tested on a test dataset (*n* = 326) that was not used during training. Performance metrics based on the holdout validation result for YOLOv5 model and the 5-fold cross-validation results for MobileNetV2, Xception and InceptionResNetV2 are presented. Statistical analysis among DNN models and clinicians was performed using the results of 35 clinicians on the test dataset. The overview of the study is shown in Fig. 2.

### Dataset

The dataset used in this retrospective study was collected and anonymized in Digital Imaging and Communications in Medicine (DICOM) format from the databases of three distinct hospitals. This is a multicenter study, which enhances the generalizability and robustness of the findings. The dataset comprises 1657 PXRs, including 699, 402, and 556 radiographic images collected from Manisa Alaşehir State Hospital, Manisa City Hospital, and Manisa Celal Bayar University Hafsa Sultan Hospital, respectively. Detailed dataset specifications are shown in Table 2 and statistics of these centers are shown in Table 3. Ethical approval was obtained from the Institutional Review Board of Manisa Celal Bayar University, Faculty of Medicine (reference number: 20.478.486/1027, no:65093, approval date: 24/11/2021). This is a retrospective study, and the dataset was anonymized. Due to the retrospective nature of the study, (the Institutional Review Board of Manisa Celal Bayar University, Faculty of Medicine) waived the need for obtaining informed consent. All methods were performed in accordance with the relevant guidelines, regulations, and ethical approval. The fracture images in the dataset were obtained from patients undergoing surgery. Each PXR image was reviewed and verified by four expert orthopedists (KG, ZOG, HKT, GO). Images (*N* = 115) exhibiting poor quality (e.g., blurry images, under/overexposure) were excluded from the dataset. To prevent overfitting and enhance the performance of deep learning models, data augmentation techniques were implemented.^30^ Specifically, an image generator was developed to enrich a batch of images using various methods, such as rotation, magnification, width-height shift, and vertical-horizontal flip. The image generator was applied with a 45-degree rotation range, a 20% zoom range, and 20% width-height shift ranges. The effectiveness of the image augmentation strategy enables the deep learning model to improve its learning performance.

**Table 2:** Dataset characteristics and patient statistics.

Characteristics	Sample size (total = 1657)
Male/female	756/901 (45.62%/54.38%)
Fracture/implant/no fracture	14.75%/12.96%/72.29
Mean Age, y	48.42 ± 30.28%
Age groups	
– < 40	666 (40.19%)
– 40–49	163 (9.84%)
– 50–59	202 (12.19%)
– 60–69	196 (11.83%)
– ≥ 70	430 (25.95%)

**Table 3:** Dataset specifications.

Data source	Fracture	Implant	No fracture	Total
Manisa Alasehir State Hospital	87	82	530	699
Manisa City Hospital	102	72	228	402
Manisa Hafsa Sultan Hospital	65	68	423	556
Total	254	222	1181	1657

### Hip area detection and fracture detection

Object detection techniques combine image classification and object localization to predict and localize different types of objects within an image. An object detection algorithm primarily involves three sequential processes: region selection, feature extraction, and classification.^31^ In this study, our dataset comprising PXR images covering a large area of patients’ bodies was collected by emergency medicine physicians to perform fracture detection using an AI model. This model aims to locate the hip region for subsequent fracture detection to achieve higher performance. The dataset for object detection training, originally in DICOM format, was converted to JPEG format, and bounding boxes for the hip regions were annotated using the labelImg toolbox.^32^ These labeled images were used to train two separate YOLOv5 models.^33^ The first YOLOv5 model was trained for an object detection-based fracture detection system. Subsequently, the second YOLOv5 model was used to identify the hip area regions within the dataset for the automated system. A new dataset containing only the hip areas for training the classification model was created. To improve the visibility of bone structures within the dataset, the CLAHE algorithm was applied.^34^ The CLAHE method adjusts the local contrast of an image by adaptively equalizing the histogram in small regions, thereby preventing over-amplification of noise. The implementation of CLAHE enhances the sharpness of bone regions, providing a clearer representation for the deep learning model. This final dataset was then utilized as input for the deep learning-based classification model.

### Patch classification

DNNs, particularly CNNs, have received significant attention for the classification of medical images in recent years due to their superior performance compared to traditional techniques.^35^ This study builds upon previous research by introducing a deep learning model in the form of an artificial neural network (ANN) designed to classify hip regions of PXR images into fracture, implant, and no-fracture classes. The ANN model comprises multiple layers, each serving a distinct function in the feature extraction and classification process. The CNN block of the DNN automatically learns the image features. The second part of the DNN classifies the learned features to produce a prediction, utilizing fully connected neural networks. The input layer of the CNN serves as the entry point for PXR images, which are subsequently processed by the convolutional layers. The weight and bias parameters of the nodes are updated with each calculation using the backpropagation technique, enabling the model to learn the features that facilitate differentiation between samples.^36^ The subsequent layer is the pooling layer, which reduces the size of the previous layer, parameters, and computational load. The flattening layer then converts the output of the CNNs into a single vector for the classification block, establishing connections between the extracted features and prediction classes. The DNN classifies samples based on these features. The final layer generates probability values for each category and produces predictions for input images.

### Transfer learning and fine tuning

Transfer learning is a machine learning approach that enables the reuse of previously trained models for new tasks, thereby reducing the amount of data and computation required and increasing performance.^37^ This method is particularly effective in deep learning, where training large-scale neural networks can be both computationally and time-intensive. Transfer learning leverages a pre-trained model, trained on a large and diverse dataset, as a starting point for a new task. The pre-trained model serves as a feature extractor, with the lower layers fine-tuned to adapt to the new task, while the higher layers remain unchanged. This is attributed to the fact that the lower layers of a neural network typically learn more general features, such as edges and shapes, whereas the upper layers learn task-specific features.

Fine-tuning is a crucial step when employing transfer learning.^38^ In this method, the entire pre-trained model is fine-tuned.

### Model implementation

Due to processing time and memory limitations, the resolution of images was reduced before being fed into the DNN. In this study, the collected images were resized to 224 × 224 × 3 pixels and 299 × 299 × 3 pixels for the deep learning models of MobileNetV2, Xception, and InceptionResNetV2. To improve the training performance of the model, the RGB values of the images were transformed to NumPy arrays and stored.

To train the deep learning models on the specific dataset, the deep learning architectures pre-trained on the ImageNet dataset were extended with additional neural network layers.^39^ Empirically, a GlobalAveragePooling layer and a Dropout layer with a ratio of 0.2 were added to improve the performance of the classification task. The output was then flattened and processed in a fully connected layer. The original fully connected layers of the ImageNet model were removed, and two 128-node dense layers, along with a dropout layer with a ratio of 0.2, were added. The final layer was a three-node output layer designed to predict fracture, implant, and no fracture. To improve the success of the pre-trained deep learning models, a two-stage training approach was used. In the initial phase, all CNN layers were frozen without the fully connected layers. This step enables the extraction of universal features from the ImageNet dataset. In the second step, all layers were included in the training and the model was fine-tuned for optimum performance by optimizing CNN nodes and universal features extracted from ImageNet. During training, a dynamic learning rate method was used to optimize the learning rate using the ReduceLROnPlateau function. If validation performance did not improve after 10 epochs, the learning rate was decreased by its 10% of its current value to ensure more effective convergence and avoid oscillations in the learning process. The minimum learning rate was set to 0.00001 to ensure a lower bound on the learning rate adjustments.

The models were trained using the Python programming language and the Keras library. The models were trained on a system that has an Nvidia RTX3090Ti graphics card with 24 GB of memory, an Intel Core i5 13600 K processor, and 64 GB of RAM.

### Statistical analysis

**Fig. 3:**
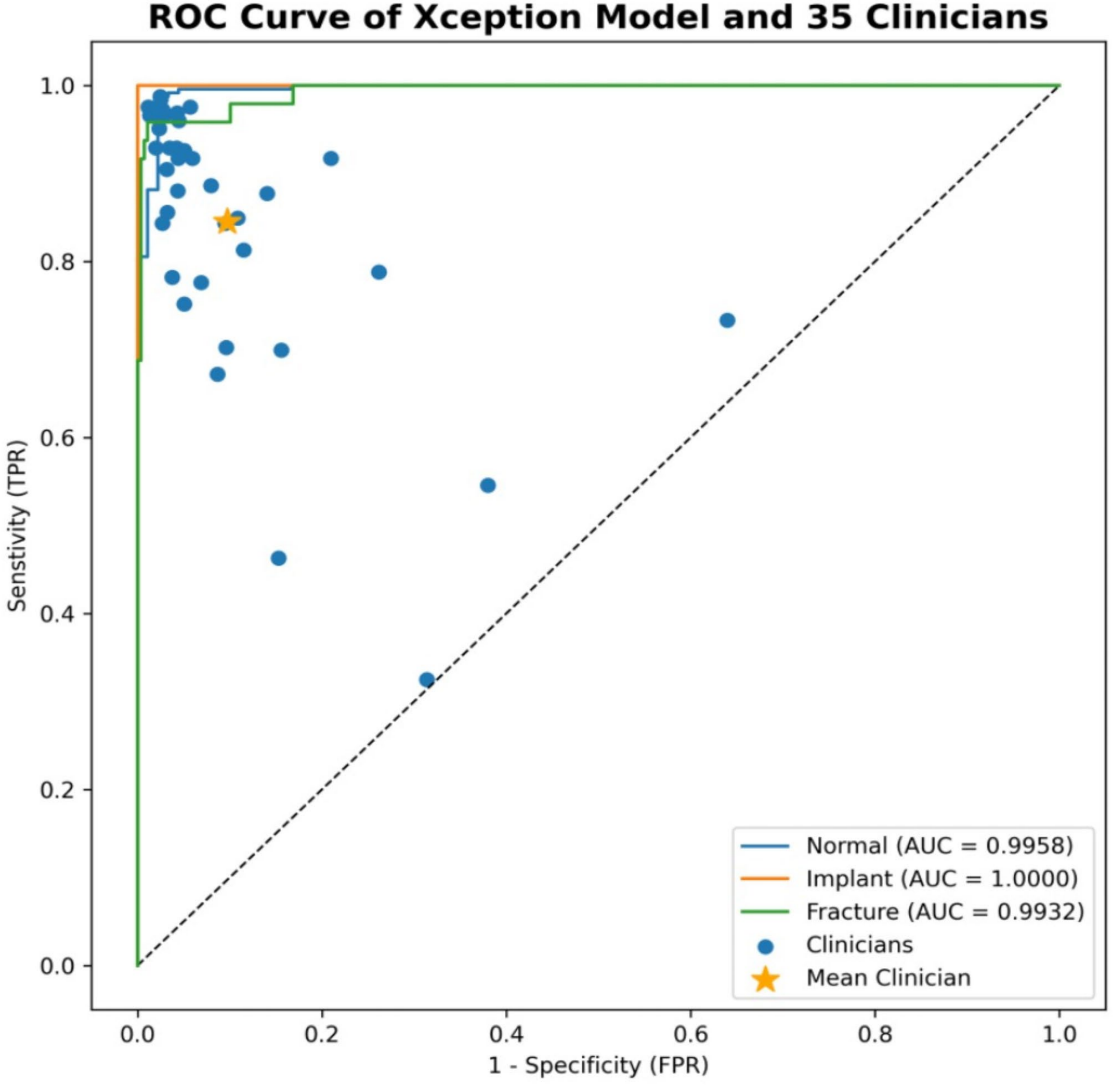
ROC Curve of the Xception model and 35 clinicians.

For object detection, the Intersection over Union (IoU) and Mean Average Precision (mAP) metrics were used to evaluate model performance. For classification performance, weighted values of accuracy, sensitivity, specificity, precision, negative predictive value (NPV), and false positive rate (FPR) for each class were calculated. To measure overall performance, the area under the receiver operating characteristic (ROC) curves were calculated as shown in Fig. 3.

For statistical significance testing, a two-sided t-test was used, with a p-value of p < 0.05  considered statistically significant to compare AI model and clinician performance. The statistical analysis was performed using Python. All values are presented as mean ± standard deviation (SD) format. For the test procedures, PXR images of the test dataset (*n* = 326) were assessed by 35 clinicians with an average of 7.24 ± 7.29 years of experience (range:1–27 years). Holdout or k-fold cross-validation and classification metrics were used to evaluate the success and reliability of the trained model. To compare the accuracy results of different AI models, a one-way ANOVA test was conducted. Furthermore, Tukey’s Honestly Significant Difference (HSD) test was also performed to measure the significance between individual models. The results of the ANOVA and HSD tests are given in Supplementary Tables 1 and 2, respectively.

## Electronic supplementary material

Below is the link to the electronic supplementary material.

Supplementary Material 1

## Data Availability

The datasets used and/or analyzed during the current study available from the corresponding author on reasonable request.

## References

[1] Pinto, A. et al. Traumatic fractures in adults: Missed diagnosis on plain radiographs in the Emergency Department. *Acta Bio Medica Atenei Parmensis* **89**, 111 (2018). 29350641 10.23750/abm.v89i1-S.7015 PMC6179080

[2] Burlew, C. C. et al. Preperitoneal pelvic packing reduces mortality in patients with life-threatening hemorrhage due to unstable pelvic fractures. *J. Trauma Acute Care Surg.* **82**, 233–242 (2017). 27893645 10.1097/TA.0000000000001324 PMC5250563

[3] Seong, Y. J., Shin, W. C., Moon, N. H. & Suh, K. T. Timing of hip-fracture surgery in elderly patients: Literature review and recommendations. *Hip Pelvis* **32**(1), 11 (2020). 32158724 10.5371/hp.2020.32.1.11 PMC7054076

[4] Sing, C. W. et al. global epidemiology of hip fractures: Secular trends in incidence rate, post-fracture treatment, and all-cause Mortality. *J. Bone Miner. Res.* **38**, 1064–1075 (2023). 37118993 10.1002/jbmr.4821

[5] Chou, C. H. et al. Hemostasis as soon as possible? The role of the time to angioembolization in the management of pelvic fracture. *World J. Emerg. Surg.* **14**, 1–8 (2019). 31210779 10.1186/s13017-019-0248-z PMC6567387

[6] Center, J. R., Nguyen, T. V., Schneider, D., Sambrook, P. N. & Eisman, J. A. Mortality after all major types of osteoporotic fracture in men and women: An observational study. *The Lancet* **353**(9156), 878–882 (1999). 10.1016/S0140-6736(98)09075-8 10093980

[7] Artoni, C. et al. Pelvic ring fractures: What about timing?. *Acta Bio Medica: Atenei Parmensis* **90**(Suppl 12), 76 (2019). 31821288 10.23750/abm.v90i12-S.8949 PMC7233695

[8] Yousef, R., Gupta, G., Yousef, N. & Khari, M. A holistic overview of deep learning approach in medical imaging. *Multimed. Syst.* **28**(3), 881–914 (2022). 35079207 10.1007/s00530-021-00884-5 PMC8776556

[9] Cheng, C. T. et al. A scalable physician-level deep learning algorithm detects universal trauma on pelvic radiographs. *Nat. Commun.* **12**(1), 1066. 10.1038/s41467-021-21311-3 (2021). 33594071 10.1038/s41467-021-21311-3 PMC7887334

[10] Krogue, J. D. et al. Automatic hip fracture identification and functional subclassification with deep learning. *Radiol. Artif. Intell.* **2**(2), 190023 (2020). 10.1148/ryai.2020190023 PMC8017394 33937815

[11] Kitamura, G. Deep learning evaluation of pelvic radiographs for position, hardware presence, and fracture detection. *Eur. J. Radiol.* **130**, 109139 (2020). 32623269 10.1016/j.ejrad.2020.109139 PMC7483754

[12] Sato, Y. et al. Artificial intelligence improves the accuracy of residents in the diagnosis of hip fractures: A multicenter study. *BMC Musculoskelet. Disord.* **22**(1), 407 (2021). 33941145 10.1186/s12891-021-04260-2 PMC8091525

[13] Lee, J. M. et al. Deep-learning-based pelvic automatic segmentation in pelvic fractures. *Sci. Rep.* **14**, 12258 (2024). 38806582 10.1038/s41598-024-63093-w PMC11133416

[14] Saeed, M. U. et al. An automated deep learning approach for spine segmentation and vertebrae recognition using computed tomography images. *Diagnostics* **13**, 2658 (2023). 37627917 10.3390/diagnostics13162658 PMC10453471

[15] Saeed, M. U., Bin, W., Sheng, J., Ali, G. & Dastgir, A. 3D MRU-Net: A novel mobile residual U-Net deep learning model for spine segmentation using computed tomography images. *Biomed. Signal Process. Control* **86**, 105153 (2023).

[16] Saeed, M. U., Bin, W., Sheng, J., Albarakati, H. M. & Dastgir, A. MSFF: An automated multi-scale feature fusion deep learning model for spine fracture segmentation using MRI. *Biomed. Signal Process. Control* **91**, 105943 (2024).

[17] Saeed, M. U. et al. An automated multi-scale feature fusion network for spine fracture segmentation using computed tomography images. *J. Imaging Informat. Med.* **37**(5), 2216–2226 (2024). 10.1007/s10278-024-01091-0 PMC11522210 38622384

[18] Caron, R., Londono, I., Seoud, L. & Villemure, I. Segmentation of trabecular bone microdamage in Xray microCT images using a two-step deep learning method. *J. Mech. Behav. Biomed. Mater.* **137**, 105540 (2023). 36327650 10.1016/j.jmbbm.2022.105540

[19] Faghani, S. et al. A deep learning algorithm for detecting lytic bone lesions of multiple myeloma on CT. *Skeletal. Radiol.* **52**, 91–98 (2023). 35980454 10.1007/s00256-022-04160-z

[20] Twinprai, N. et al. Artificial intelligence (AI) vs. human in hip fracture detection. *Heliyon*10.1016/j.heliyon.2022.e11266 (2017). 10.1016/j.heliyon.2022.e11266 PMC9634369 36339768

[21] Reichert, G. et al. How can a deep learning algorithm improve fracture detection on X-rays in the emergency room?. *J. Imaging* **7**(7), 105. 10.3390/jimaging7070105 (2021). 39080893 10.3390/jimaging7070105 PMC8321374

[22] Liu, F. Y. et al. Automatic hip detection in anteroposterior pelvic radiographs—A labelless practical framework. *J. Personal. Med* **11**(6), 522. 10.3390/jpm11060522 (2021). 10.3390/jpm11060522 PMC8226859 34200151

[23] Selvaraju, R. R., et al. Grad-cam: Visual explanations from deep networks via gradient-based localization. In *Proceedings of the IEEE International Conference on Computer* (2017).

[24] Jiménez-Sánchez, A. et al. Precise proximal femur fracture classification for interactive training and surgical planning. *Int. J. Comput. Assist. Radiol. Surg.* **15**, 847–857 (2020). 32335786 10.1007/s11548-020-02150-x

[25] Jiang, L. et al. Opportunities and challenges of artificial intelligence in the medical field: Current application, emerging problems, and problem-solving strategies. *J. Int. Med. Res.* **49**, 03000605211000157 (2021). 33771068 10.1177/03000605211000157 PMC8165857

[26] Razai, M. S. et al. Implementation challenges of artificial intelligence (AI) in primary care: Perspectives of general practitioners in London UK. *PLoS ONE* **19**, e0314196 (2024). 39570873 10.1371/journal.pone.0314196 PMC11581230

[27] Ahmed, M. I. et al. A systematic review of the barriers to the implementation of artificial intelligence in healthcare. *Cureus* **15**, e46454 (2023). 37927664 10.7759/cureus.46454 PMC10623210

[28] Kelly, C. J., Karthikesalingam, A., Suleyman, M., Corrado, G. & King, D. Key challenges for delivering clinical impact with artificial intelligence. *BMC Med.* **17**, 1–9. 10.1186/s12916-019-1426-2 (2019). 31665002 10.1186/s12916-019-1426-2 PMC6821018

[29] Pouyanfar, S. et al. A survey on deep learning: Algorithms, techniques, and applications. *ACM Comput. Surv. (CSUR)* **51**(5), 1–36 (2018).

[30] Perez, L.,& J. W. The effectiveness of data augmentation in image classification using deep learning. http://arxiv.org/abs/1712.04621 (2017).

[31] Zhao, Z. Q., Zheng, P., Xu, S. T. & Wu, X. Object detection with deep learning: A review. *IEEE Trans. Neural Netw. Learn. Syst.* **30**(11), 3212–3232 (2019). 30703038 10.1109/TNNLS.2018.2876865

[32] HumanSignal/labelImg. https://github.com/HumanSignal/labelImg.

[33] ultralytics/yolov5: v7.0—YOLOv5 SOTA Realtime Instance Segmentation. 10.5281/ZENODO.7347926.

[34] Pizer, S. M., Johnston, R. E., Ericksen, J. P., Yankaskas, B. C. & Muller, K. E. Contrast-limited adaptive histogram equalization: Speed and effectiveness stephen m. pizer, r. eugene johnston, james p. ericksen, bonnie c. yankaskas, keith e. muller medical image display research group. In* Proceedings of the First Conference on Visualization in Biomedical Computing* (1990).

[35] Litjens, G. et al. A survey on deep learning in medical image analysis. *Med. Image Anal.* **42**, 60–88 (2017). 28778026 10.1016/j.media.2017.07.005

[36] He, K., Zhang, X., Ren, S., & Sun, J. Deep residual learning for image recognition. In *Proceedings of the IEEE Conference on Computer Vision and Pattern Recognition* (2016).

[37] Pan, S., et al. A survey on transfer learning. *IEEE Trans. Knowl. Data Eng.* (2009).

[38] Yosinski, J., et al. How transferable are features in deep neural networks? In *Advances in Neural Information Processing Systems *(2014).

[39] Krizhevsky, A., et al. Imagenet classification with deep convolutional neural networks. In *Proceedings* (2012).

