# Supplementary material for: An automated hip fracture detection, classification system on pelvic radiographs and comparison with 35 clinicians
Source: Sci Rep. 2025 May 8;15:16001. doi: 10.1038/s41598-025-98852-w (PMC12062471; doi:10.1038/s41598-025-98852-w)
Supplement: Supplementary file 1 — Supplementary Material 1 [file 41598_2025_98852_MOESM1_ESM.pdf]

Supplementary Materials

An Automated Hip Fracture Detection, Classification System on Pelvic Radiographs and Comparison with 35 Clinicians

Abdurrahim Yilmaz[1,*], Kadir Gem[2], Mucahit Kalebasi[3], Rahmetullah Varol[3], Zuhtu Oner Gencoglan[4], Yegor Samoylenko[3], Hakan Koray Tosyali[5], Guvenir Okcu[5], Huseyin Uvet[3]

Statistical Analysis

To compare the results of different AI models, a one-way ANOVA test was conducted. Use of the ANOVA test was justified by the Shapiro-Wilk test for normality (p=0.76, p=0.75, p=30, p=0.54, p=0.98, and p=0.24 for MobileNetV2 C - NC, Xception C - NC, and InceptionResNetV2 C – NC models respectively) and Levene’s test for homogeneity of variance (p=0.12). Furthermore, Tukey’s Honestly Significant Difference (HSD) test was also conducted to measure the significance between individual models. The results of the ANOVA and HSD tests are given in Supplementary Table 1 and 2 respectively.

Supplementary Table 1: Results for one way ANOVA test comparing the accuracy results between all models. Pr(>F) < 0.05 indicates significant difference in at least one model's accuracy.

	Sum of Squares	Degrees of Freedom	F	Pr(>F)
C	39.57	5	11.03	0.000013
Residual	17.21	24		

Supplementary Table 2: Tukey’s Honestly Significant Difference (HSD) test results. Results indicate that while the MobileNetV2 models accuracies are significantly lower, there is no significant difference between Xception and InceptionResNetV2 models.

Group 1	Group 2	Mean difference	Adjusted p	Lower threshold	Upper threshold	Reject
MobileNetV2 – C	MobileNetV2 – NC	0.5105	0.9281	-1.1456	2.1665	False
MobileNetV2 – C	Xception – C	1.9721	0.0132	0.316	3.6282	True
MobileNetV2 – C	Xception – NC	3.1392	0.0001	1.4831	4.7952	True
MobileNetV2 – C	InceptionResNetV2 – C	2.7138	0.0005	1.0578	4.3699	True
MobileNetV2 – C	InceptionResNetV2 – NC	2.3941	0.002	0.7381	4.0502	True
MobileNetV2 – NC	Xception – C	1.4616	0.1059	-0.1944	3.1177	False
MobileNetV2 – NC	Xception – NC	2.6287	0.0007	0.9727	4.2848	True
MobileNetV2 – NC	InceptionResNetV2 – C	2.2034	0.0047	0.5473	3.8594	True
MobileNetV2 – NC	InceptionResNetV2 – NC	1.8837	0.0194	0.2276	3.5397	True
Xception – C	Xception – NC	1.1671	0.2835	-0.489	2.8232	False
Xception – C	InceptionResNetV2 – C	0.7417	0.7353	-0.9143	2.3978	False
Xception – C	InceptionResNetV2 – NC	0.422	0.9669	-1.234	2.0781	False
Xception – NC	InceptionResNetV2 – C	-0.4254	0.9658	-2.0814	1.2307	False
Xception – NC	InceptionResNetV2 – NC	-0.7451	0.7318	-2.4011	0.911	False
InceptionResNetV2 – C	InceptionResNetV2 – NC	-0.3197	0.9903	-1.9758	1.3364	False

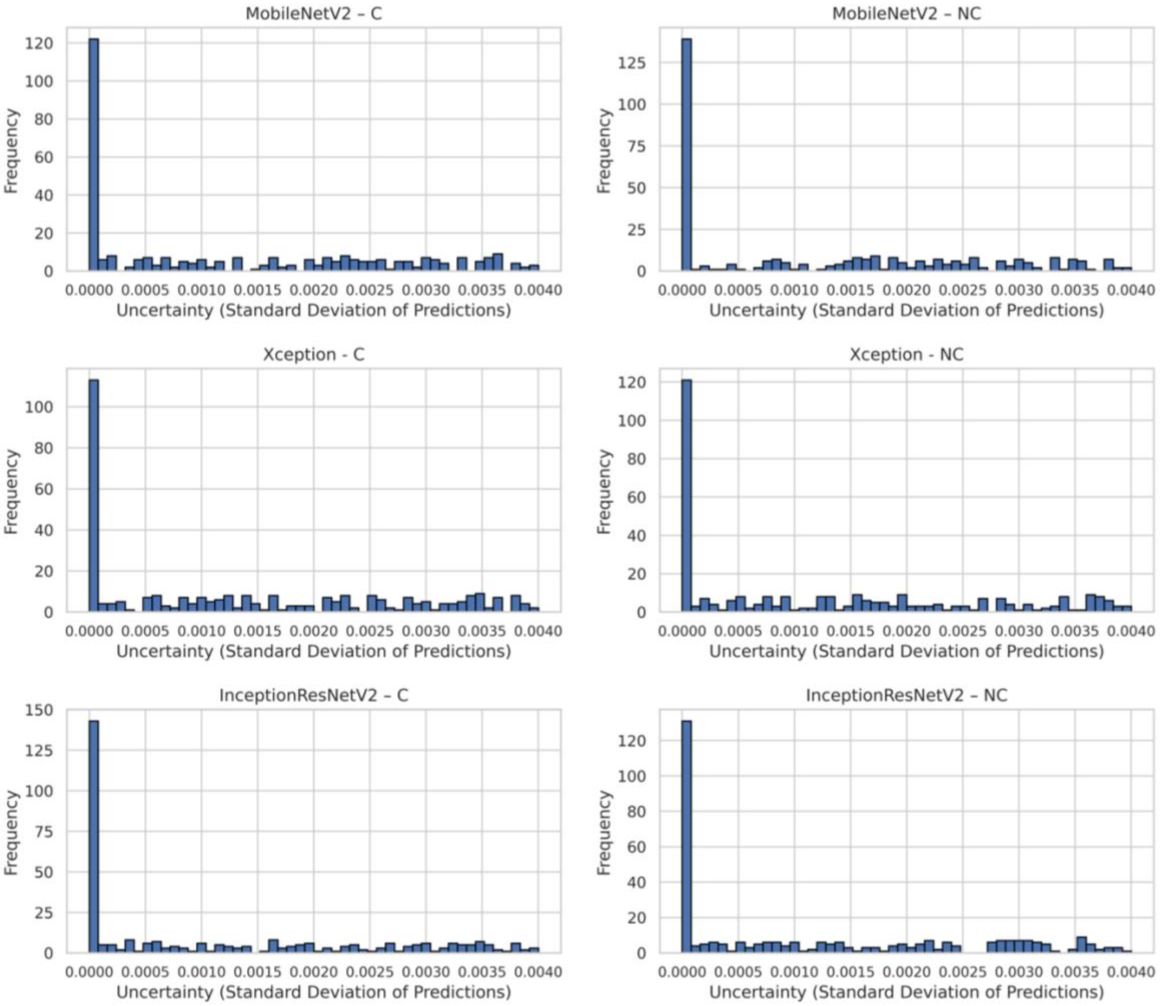

Supplementary Fig. 1. Histogram of uncertainty values for each model calculated using the Monte Carlo dropout method. Standard deviation values were calculated from 200 iterations of forward-passes with random dropouts at each iteration. Significant number of test samples showed near-zero variation between iterations. This is due to these samples being assigned to the same class with high accuracy.
